# Antibacterial Activity and Epigenetic Remodeling of Essential Oils from Calabrian Aromatic Plants

**DOI:** 10.3390/nu14020391

**Published:** 2022-01-17

**Authors:** Patrizia D’Aquila, Ersilia Paparazzo, Michele Crudo, Sonia Bonacci, Antonio Procopio, Giuseppe Passarino, Dina Bellizzi

**Affiliations:** 1 Department of Biology, Ecology and Earth Sciences, University of Calabria, 87036 Rende, Italy; patrizia.daquila@unical.it (P.D.); ersilia.paparazzo@unical.it (E.P.); michelecrudo22@gmail.com (M.C.); giuseppe.passarino@unical.it (G.P.); 2 Department of Health Sciences, University Magna Graecia of Catanzaro, 88100 Catanzaro, Italy; s.bonacci@unicz.it (S.B.); procopio@unicz.it (A.P.)

**Keywords:** essential oils, nutrition, herbs, spices, antimicrobial, MIC, MBC, cytosine methylation, adenine methylation, antibiotic resistance

## Abstract

Natural compounds have historically had a wide application in nutrition. Recently, a fundamental role has been identified for essential oils extracted from aromatic plants for their nutritional, antimicrobial, and antioxidant properties, and as food preservatives. In the present study, essential oils (EOs) from ten aromatic plants grown in Calabria (Italy), used routinely to impart aroma and taste to food, were evaluated for their antibacterial activity. This activity was investigated against *Escherichia coli* strain JM109, and its derived antibiotic-resistant cells selected by growing the strain at low concentrations of ampicillin, ciprofloxacin, and gentamicin by measuring the minimum inhibitory concentration (MIC) and the minimum bactericidal concentration (MBC). Although all the essential oils showed bactericidal activity, those from *Clinopodium nepeta*, *Origanum vulgare*, and *Foeniculum vulgare* displayed the greatest inhibitory effects on the bacterial growth of all cell lines. It is plausible that the antibacterial activity is mediated by epigenetic modifications since the tested essential oils induce methylation both at adenine and cytosine residues in the genomes of most cell lines. This study contributes to a further characterization of the properties of essential oils by shedding new light on the molecular mechanisms that mediate these properties.

## 1. Introduction

Herbs and spices have been used from the beginning of human history as an essential part of human nutrition and for their beneficial properties [1,2,3]. The consumption of herbs and spices is an important aspect of the traditional Mediterranean diet, and, along the human history of every culture, they have been used to add flavor and aroma to dishes and as food preservatives [4,5,6]. They were also used in cosmetics to mask unpleasant odors or to attract the attention of other people, and in medicine due to their septic, analgesic, and anti-inflammatory properties [4,7,8,9].

Essential oils (EOs) are volatile secondary metabolites of aromatic plants and spices that give them their characteristic and distinctive smell or taste [10,11,12]. EOs are generally extracted by water vapor distillation (hydro distillation), steam distillation or dry distillation starting from fresh or dry plant raw materials; an exception is Eos derived from the *Citrus genus*, which are usually extracted by mechanical cold pressing of the fruit peel [13,14,15].

EOs are produced by more than 18,000 species of plant, including many gymnosperm and angiosperm families; among them, only 250–300 EOs are produced and commercialized. Depending on the plant species, EOs are produced and stored in the different plant tissues in complex secretory structures, such as glands, secretory cavities, and resin conduits. EOs are synthesized through the pathways of malonic acid, mevalonic acid, and methyl-d-erythritol-4-phosphate (MEP) in the cytoplasm and plastids of specific plant cells. EOs are very complex mixtures of volatile organic macromolecules; mainly terpenes, terpenoids, and phenylpropanoids, but they may also contain other compounds, such as oxide, sulfur derivatives and fatty acids [16]. However, the total composition of each EO could be much more complex and can reach more than 300 different compounds. In nature, EOs play an important ecological role for plants, including intra- and inter-species plant communication, repellent and deterrent activities against insects and predators, pollinator attraction, the inhibition of seed germination, and antibacterial, antifungal, and wound healing activities [17].

Foodborne diseases and food spoilage due to microbial contamination are a growing public health problem worldwide [18,19]. Furthermore, the extensive use of antimicrobial products in humans and animal farming has greatly contributed to the selection of resistant bacterial strains [20,21]. Natural and organic compounds, such as EOs, are becoming one of the most promising research topics for their applications in food and nutraceutical products, as an increased amount of research has pointed out their beneficial effects on health with little or no side effects, and they are cost effective and environmentally friendly when compared with non-organic synthetic compounds [22]. Therefore, plant-derived natural antibacterial and antimycotics substances are becoming new green and ideal alternatives to chemical preservatives in the food industry and are powerful potential therapeutic tools [23,24]. Several EOs have demonstrated a well-characterized antimicrobial activity against both Gram-positive and Gram-negative bacteria; furthermore, some EOs are also active against other microorganisms, such as viruses and yeast [25,26,27,28,29]. Different mechanisms of action seem to be involved in Eos’ antibacterial actions, such as irreversible damage of the bacterial cell wall and membrane, the inhibition of metabolic pathways and protein synthesis, and interference with cell wall synthesis and DNA and RNA synthesis [24,30,31]. Moreover, several EOs seem to be able to modulate the virulence of some bacterial strains by inhibiting bacterial cell communication, biofilm formation and toxin production and by modulating the expression of virulence genes and the quorum sensing system [7,32,33,34,35].

This study aimed to evaluate whether ten essential oils extracted from aromatic plants grown in Calabria (Italy) and their major components exert antimicrobial activity on *Escherichia coli* strain JM109 and three lines derived by growing it at low concentrations of ampicillin, ciprofloxacin, and gentamicin. Epigenetic modifications induced by the essential oils were also investigated.

## 2. Materials and Methods

### 2.1. Bacterial Strains and Growth Conditions

This study was carried out on *Escherichia coli* strain JM109 (Stratagene, La Jolla, CA, USA) (e14–(McrA–) recA1 endA1 gyrA96 thi-1 hsdR17 (rK– mK+) supE44 relA1 Δ (lac-proAB) [F’ traD36 proAB lacIqZΔM15]) and ampicillin- (Amp^r^), ciprofloxacin- (Cip^r^), and gentamicin- (Gen^r^) resistant cells. These cell lines were obtained by growing the parental JM109 cells at low concentrations of the three antibiotics following the procedure described by Sandoval-Motta and Aldana, 2016 [36]. The bacterial strains were kept frozen in stock cultures at −80 °C in cryovials.

### 2.2. Essential Oils (EOs) Extraction

The vegetable raw material of 10 plant species was collected from wild areas and local organic farmers located in Calabria. The following species were selected for the extraction of the essential oils (EOs): *Clinopodium nepeta* (L.) *Kuntze*, *Citrus bergamia*, *(Risso & Poit.)*, *Citrus limon* (L.) *Osbeck*, *Citrus reticulata (Blanco)*, *Foeniculum vulgare* subsp. *piperitum (Ucria) Bég.*, *Laurus nobilis* L., *Myrtus communis* L., *Origanum vulgare* L. subsp. *viridulum (Martrin-Donos) Nyman*, *Salvia officinalis* L., and *Salvia rosmarinus Spenn*.

For the essential oils extraction were used the fruit peel of *Citrus bergamia (Risso & Poit.)* and *Citrus limon* (L.) *Osbeck*, the flower, leaf, and terminal branches of *Clinopodium nepeta* (L.) *Kuntze*, *Foeniculum vulgare* subsp. *piperitum (Ucria) Bég.*, *Myrtus communis* L., *Origanum vulgare* L. subsp. *viridulum (Martrin-Donos*) *Nyman*, *Salvia officinalis* L. and *Salvia rosmarinus Spenn*, while for *Citrus reticulata (Blanco*) and *Laurus nobilis* L. only the leaf and terminal branches were used. The essential oil of *Citrus bergamia (Risso & Poit.*) was mechanically extracted by a local producer by industrial cold expression process from fresh fruit. For all the other species the essential oils were extracted by the water vapor under-vacuum distillation process in a 20 L inox apparatus, starting for fresh collected raw material from a local essential oil producer. The essential oils were aliquoted and kept in dark glass bottles, tightly sealed at +4 °C, until use.

### 2.3. Analysis of Chemical Composition of Essential Oils

GC-–MS (Gas Chromatography-Mass Spectrometry) analyses were performed using a gas chromatograph (Focus GC-Thermo Fisher, Milan, Italy) equipped with a Varian VF-5m (30 m × 0.25 mm × 0.25 μm) capillary column, combined with a single quadrupole mass spectrometer (DSQII-Thermo Scientific, Milan, Italy). The samples were diluted 1:1000 in ether. One microliter of sample was injected in spitless mode at a temperature of 220 °C. The column flow rate was 1 mL min^−1^ using helium as carrier gas. The initial GC oven temperature was 55 °C, increased by 4 °C min^−1^ to 240 °C with a hold time of 3 min. The transfer line temperature was 250 °C. The MS was operated using electron impact (EI) at an ionization energy of 70 eV. The ion source temperature was set at 250 °C. The solvent delay for the mass spectrometry was set at 3 min and the EI scan mode was used for identification, covering the range of 25–350 *m/z*. The compound was identified by comparison with the NIST database (https://www.nist.gov/pml/atomic-spectra-database, accessed on 6 June 2021).

The instrumentation performance, chromatograms, mass spectra and initial data processing were carried out with the supplied Xcalibur software (Thermo Fisher, Milan, Italy).

### 2.4. Determination of Minimum Inhibitory Concentration (MIC) and Minimum Bactericidal Concentration (MBC)

The minimum inhibitory concentration (MIC) of the essential oils on the four bacterial cell lines was determined by the broth dilution method, carried out in sterile glass tubes.

Since EOs are highly lipophilic organic mixtures, inulin powder (CAS. N. 9005-80-5, Farmalabor SRL, Canosa di Puglia BT, Italy) was used as carrier. The working solutions were prepared daily by letting the oil adsorb to the inulin (100 µL EOs/gr inulin) by vigorous agitation at regular intervals for at least 90 min at room temperature, and its subsequent dissolution in LB medium. Approximately 10^7^ cells from an overnight LB culture of each cell line were inoculated into tubes containing 3 mL of the following serial dilutions of the dissolved EOs: 0.1, 0.2, 0.3, 0.4, 0.5, 0.6, 0.7, 0.8, 0.9, 1, 2, 3, 4, 5, 6, 7, 8, 9, and 10 µL of EO/mL of medium. Culture tubes were shaken at 300 rpm at 37 °C for 18 h.

In all experiments, medium with (positive control) and without (negative control) cells and free of EOs were also analyzed to check the adequacy of microbial growth and sterility, respectively. In addition, two further controls were represented by cell-free medium in the presence of each EO to discern the turbidity background, as well as by medium-containing cells in the presence of the different inulin dilutions but free of Eos, to evaluate its potential effect on bacterial growth.

Turbidity measurement was performed at 600 nm in a spectrophotometer. MIC values were determined as the lowest concentration of essential oil corresponding to values of optical density (OD) comparable to those of cell-free liquid Luria-Bertani (LB) medium. Minimum bactericidal concentration (MBC) values were calculated by subculture of all dilutions carried out in liquid medium on agar plates. The MBC was determined by considering the lowest concentration of EO which reduces the viability of the initial bacterial inoculum by ≥99.9%. Each experiment was carried out in triplicate, with three independent repetitions.

### 2.5. DNA Extraction

Genomic DNA was extracted from untreated bacterial cells as well as from cells under pre-inhibitory concentrations (pre-MICs) of EOs by using a DNeasy UltraClean Microbial Kit (Qiagen, Milan, Italy) according to the manufacturer’s protocol. Briefly, 3 mL pellets of bacterial cell culture were suspended in 300 µL of PowerBead Solution and vortexed. Resuspended cells were transferred to PowerBead Tubes and then 50 µL of Solution SL was added. After vortex for 10 min, the tubes were centrifuged at 10,000× *g* for 30 s. A total of 100 µL of Solution IRS was added to the supernatants and incubated at 4 °C for 5 min. After a centrifugation at 10,000× *g* for 1 min, 900 µL of Solution SB was added to the supernatants. Subsequently, 700 µL of sample was loaded into MB Spin Columns and centrifuged at 10,000× *g* for 30 s. The centrifugation was repeated after adding 300 µL of Solution CB and the flow-through discarded. DNA samples were eluted by a centrifugation at 10,000× *g* for 30 s in 50 µL of Solution EB.

The DNA concentration and purity were determined spectrophotometrically, and purity of the sample evaluated using the 260/280 nm absorbance ratio.

### 2.6. Quantification of Global 5-Methylcytosine and N6-Methyladenosine Levels

Global DNA methylation levels of 5-methylcytosines (5mC) and N6-methyladenosines (m6A) were determined by using the MethylFlash Global DNA Methylation (5mC) ELISA Easy Kit and MethylFlash m6A DNA Methylation ELISA Kit (Epigentek, Farmingdale, Nassau County, NY, USA), respectively, following the manufacturer’s instructions. Shortly, the methylated fraction of bacterial genomic DNA, through ELISA-like reactions, was recognized by the 5mC or m6A antibodies and quantified in a microplate spectrophotometer by reading the absorbance at 450 nm.

In each experiment, the percentage of 5mC and m6A was calculated using the second-order regression equation of a standard curve that was constructed by mixing equivalent molar concentrations at different ratios of full unmethylated and methylated control DNA. Each sample was analyzed in triplicate. The methylation values of each untreated cell line were used as reference values (relative quantification) for the corresponding cell line treated with essential oil.

### 2.7. Statistical Analysis

Statistical analyses were performed using SPSS 20.0 statistical software (SPSS Inc., Chicago, IL, USA). One-way analysis of variance (ANOVA) and Student’s *t*-test were adopted. Significance level was defined as *p* ≤ 0.05.

## 3. Results

### 3.1. Antibacterial Activity of Essential Oils

The in vitro antibacterial activity of ten EOs on the Gram-negative *Escherichia coli* strain JM109 and on resistant cells to ampicillin (Amp^r^), ciprofloxacin (Cip^r^), and gentamicin (Gen^r^), selected by the growth of the parental line at low concentrations of antibiotic, was evaluated by determining the MIC and the MBC values. The results obtained are reported in Table 1. These assays revealed that all the essential oils analyzed show bactericidal activity, as deduced by MIC values, and confirmed by MBC. EOs from *Clinopodium nepeta*, *Origanum vulgare* and *Foeniculum vulgare* showed the greatest inhibitory effect on bacterial growth. In fact, very low MIC values were identified in both the parental cell line and the three antibiotic-resistant cells, showing a spectrum of activity ranging from 0.300 to 0.966 µL/mL. Conversely, the antibacterial activity of *Citrus bergamia*, *Citrus limon*, *Myrtus communis* and *Salvia officinalis* was less effective in all cell lines since they exhibited high MIC values. However, as can be seen, the activity of the four essential oils in the three antibiotic-resistant cell lines shows variability with respect to the parental line, both greater, as in the case of *Citrus bergamia* in Gen^r^ and Cip^r^ (10 µL/mL), *Citrus limon* in Cip^r^ (8.333 µL/mL) and *Myrtus communis* in Cip^r^ (10 µL/mL), and less, as in *Citrus bergamia* in Amp^r^ (4.667 µL/mL), in *Citrus limon* in Amp^r^ (2.000 µL/mL) and Gen^r^ (3.000 µL/mL) and in *Salvia officinalis* in Amp^r^ (2.000 µL/mL), Gen^r^ (4.000 µL/mL) and Cip^r^ (4.333 µL/mL). Intermediate MIC values, corresponding to concentrations of essential oils ranging from 2.000 to 3.000 µL/mL, were observed following treatment with *Citrus reticulata*, *Laurus nobilis*, and *Salvia rosmarinus* in all cell lines. The only exception with a higher value of MIC is observable for *Salvia rosmarinus* in Cip^r^ (6.333 µL/mL). These effects could be related to the presence of the tested essential oils, identified by gas chromatography–mass spectrometry (GC–MS) analysis and listed in Table 2. Each essential oil presents a unique and characteristic terpenic profile. It was possible to observe that the essential oil of *Origanum vulgare*, belonging to subspecies *viridulum*, a unique autochthonous plant typical of South Italy, was characterized by a high level of p-thymol (47.31%), followed by terpinene and p-cymene (18.52% and 11.78%, respectively). On the other hand, the EO of *Clinopodium nepeta* was characterized by high levels of piperitenone oxide (18.23%), (+)-limonene (15.80%) and (+)-pulegone (13.75%), while the EO of *Foeniculum vulgare* was mainly composed of estragole (45.33%), α-pinene (14.71%), anethal (14.54%) and fenchone (11.24%).

### 3.2. Effects of EOs on the Methylation Profiles of Citosine and Adenine

**Figure 1:**
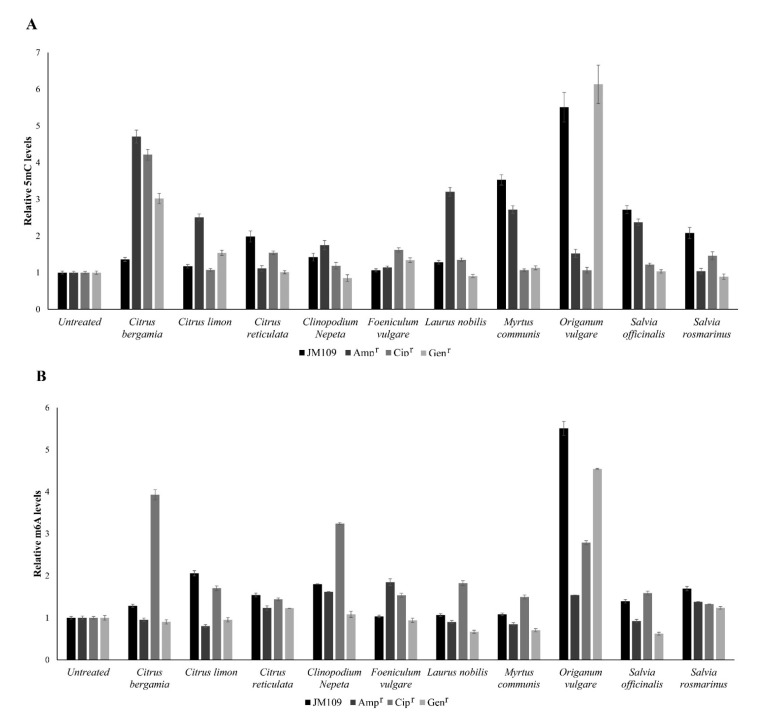
Methylation levels of 5-methylcytosine (5mC) (**A**) and N6-methyladenosine (m6A) (**B**) residues in DNA samples extracted from the *Escherichia coli* JM109 strain, and ampicillin-, ciprofloxacin-, and gentamicin-resistant cell lines at basal conditions (untreated) and after treatment with pre-MIC concentrations of the essential oils. The values represent the mean of three independent triplicate experiments with standard error.

The global methylation levels of cytosine and adenine residues were evaluated in DNA samples extracted from the JM109 and antibiotic-resistant cell lines, kept in culture in the absence and the presence of each essential oil at the concentrations corresponding to the pre-MIC values. The abundance of 5-methylcytosine (5mC) and N6-methyladenosine (m6A) is reported in Figure 1.

By comparing the cell lines before and after treatment with the essential oils, we can observe that it induces a general up methylation of the cytosine residues in all four cell lines (*p* < 0.05), with some exceptions. Specifically, no significant change is observed in the methylation status of cytosines in Cip^r^ treated with *Citrus limon*, *Origanum vulgare*, and *Myrtus communis*, as well as in the Gen^r^ line treated with *Citrus reticulata*, *Clinopodium nepeta*, *Laurus nobilis*, *Salvia officinalis*, and *Salvia Rosmarinus*. Similarly, neither the treatment with *Foeniculum vulgare* against JM109 nor with that of *Citrus reticulata* and *Salvia Rosmarinus* against Amp^r^ cell lines induced significant changes in the methylation levels of cytosines (Figure 1A).

By comparing the response to EO treatment of the antibiotic-resistant lines with that of the parental line, all three antibiotic-resistant lines exhibit significant up methylation compared with the JM109 parental cell line after treatment with *Citrus bergamia*. A similar trend can also be observed with *Foeniculum vulgare*, but only for the Cip^r^ and Gen^r^ lines, and with *Citrus limon* for Amp^r^ and Gen^r^ lines, as well as following treatment *with Clinopodium nepeta* and *Laurus nobilis* for the sole Amp^r^ cell line. Conversely, lower levels of 5mC were observed in all the antibiotic-resistant lines than the JM109 parental cell line in response to treatment with *Citrus reticulata*, *Myrtus communis*, *Salvia officinalis*, and *Salvia rosmarinus*. Similarly, the same situation was observed in the Amp^r^ and Cip^r^ cell lines after treatment with *Origanum vulgare* as well as in the Gen^r^ cell line after treatment with *Clinopodium nepeta* and *Laurus nobilis* (Figure 1A).

More variability in the response to EO treatment was observed among the four cell lines regarding the methylation levels of adenine residues (Figure 1B). An increase in the m6A levels was observed in all cell lines following treatment with *Citrus reticulata*, *Origanum vulgare*, and *Salvia Rosmarinus*; meanwhile, a decrease was noticed in both the Amp^r^ cells treated with *Citrus limon* and *Myrtus communis* and in the Gen^r^ cells treated with *Laurus nobilis*, *Myrtus communis*, and *Salvia officinalis*. No change in m6A levels was observed in the JM109 parental cell line treated with *Foeniculum vulgare*, *Laurus nobilis* and *Myrtus communis*, in the Amp^r^ cells treated with *Citrus bergamia* and *Salvia officinalis*, or in the Gen^r^ cells treated with *Citrus bergamia*, *Citrus limon*, *Foeniculum vulgare* and *Clinopodium nepeta*. Furthermore, the m6A levels of the three antibiotic-resistant lines following treatment with all essential oils has always been different from the parental line JM109 (*p* < 0.05) except for Gen^r^ cells treated with *Foeniculum vulgare.*

## 4. Discussion

In recent years, natural extracts have been emerging as valid alternatives to equivalent synthetic compounds, finding wide application in the food, aromatherapy, and nutraceutical industries, as well as in the clinical field. In this context, the antibacterial properties of a variety of essential oils are widely described. To date, however, no evidence has yet been reported regarding their potential role in the regulation of bacterial epigenetic profiles.

To this purpose, we evaluated the antimicrobial activity of essential oil extracts from ten aromatic plants grown in Calabria, *Clinopodium nepeta*, *Citrus bergamia*, *Citrus limon*, *Citrus reticulata*, *Foeniculum vulgare*, *Laurus nobilis*, *Myrtus communis*, *Origanum vulgare*, *Salvia officinalis*, and *Salvia rosmarinus*. They are all plants that can be used as condiments or eaten, and are important for their nutritional profile [37,38]. Due to the presence of compounds with antibacterial and antioxidant activity, they can be also used in the food industry as preservatives to prevent the spoilage of the products and to increase their shelf life.

The hydrophobic nature of essential oils involves the adoption of different methods of solubilization and delivery systems to increase their solubility in water. Here, we availed of inulin, a polyfructans dietary fiber, widely used as prebiotic and as a preservative in the food industry [39,40].

Our study revealed that all essential oils possess antibacterial activity against the *Escherichia coli* JM109 strain, and three lines selected by growing this strain at low concentrations of ampicillin, ciprofloxacin, and gentamicin antibiotics.

The most effective oils were those obtained from *Clinopodium nepeta*, *Origanum vulgare* and *Foeniculum vulgare*; meanwhile, a limited activity was exhibited by those from *Citrus bergamia*, *Citrus limon*, *Myrtus communis*, and *Salvia officinalis*. In particular, the antimicrobial activity of *Origanum vulgare* is consistent with previous studies that have shown its efficacy not only in *Escherichia coli* but also in a variety of other bacterial and fungal species, including *Pseudomonas aeruginosa*, *Staphylococcus aureus*, *Listeria monocytogenes*, *Salmonella typhimurium*, *Penicilium chrysogenum*, *Alternaria alternata*, and *Chaetomium globosum* [41,42]. Similarly, the antibacterial properties of *Clinopodium nepeta* have been previously demonstrated in both Gram-positive and Gram-negative pathogenic bacteria [43,44]. The antimicrobial efficacy we observed for *Foeniculum vulgare* is in line with the effects described by Dadalioglu and Evrendilek (2004) on the foodborne pathogenic bacteria *Escherichia coli O157:H7*, *Staphylococcus aureus*, *Listeria monocytogenes*, and *Salmonella* *typhimurium*, and by Ruberto and coll. (2000), which proved its degree of growth inhibition against a series of animal and plant pathogens, food poisoning and spoilage bacteria [45,46]. Despite these data, other evidence seems to disagree with our results [47,48]. Many aspects may play roles in these variations, including the specificity of the parts of the plant from which the oils are extracted and the chemical characteristics of essential oils and, thus, their biological properties. For example, we examined *Origanum vulgare*, which belongs to the high-yielding, thymol-type biotypes, with thymol, γ-terpinene, and p-cymene as three main components, unlike other studies analyzing biotypes with high content in carvacrol [49]. In addition, several environmental factors must be considered that influence the nature of oils, such as altitude, temperature, harvest season, and geographical position [50,51,52,53].

To our knowledge, this is the first study that demonstrates how the antibacterial activity of essential oils can be exerted also against antibiotic-resistant cell lines. Indeed, if until now the antibacterial activity of oils has been assayed on a wide range of microorganisms, it has never been tested on resistant ones. Here, we focused our attention on resistant cells selected at sub-MIC concentrations of antibiotics. The analysis of these resistant cell lines is of note, as gradually emerging is the importance of the selection of resistance to low levels of antibiotics, and not only the selection of resistance occurring at high therapeutic levels of antibiotics. During antibiotic treatment, concentrations in the body can undergo significant variations during the treatment and between different body compartments, regardless of the high doses administered [54]. As a result, treatment can select cells resistant to low levels of the antibiotic. In the external environment, the concentrations of antibiotics due to both production by microorganisms and human contamination are generally very low. Therefore, the condition of the exposure of bacteria to low concentrations of antibiotics can be a characteristic of many different environments and contexts and represents a source of the spread of bacterial resistance both in the food and environmental fields.

This evidence leads us to suggest that the essential oils we analyzed could be administrated simultaneously with classic antibiotics to limit or counteract the development of antibiotic resistance. For this potential application, toxicological studies in a mammalian system need to be further investigated. Furthermore, since EOs extracted from plants are widely used as flavoring and as food, the antibacterial effects they exert could result in beneficial effects at the level of the gastrointestinal tract, counteracting the proliferation of potentially pathogenic microorganisms or balancing situations of dysbiosis [55].

A further strength of our study is represented by having determined that the antibacterial properties of EOs are mediated by epigenetic modifications of the bacterial genomes, thus shedding light on the molecular mechanisms through which EOs act at the intracellular level, which have so far been poorly understood. In most cases, we observed up methylation at both cytosine and adenine residues after treatment with EOs. Whether this is related to the inhibition or activation of gene expression will be the subject of future studies. To date, in fact, it has not yet been demonstrated whether DNA methylation in bacteria is primarily associated with gene silencing, as in eukaryotes [56].

The results obtained in this study open new scenarios in the evaluation of the role of EOs in different fields, spanning from the environmental and microbial to the nutritional and clinical, which can lead to innovative food preparations with functional properties.

## Figures and Tables

**Table 1:** Minimal inhibitory concentrations (MIC) and minimum bactericidal concentrations (MBC) expressed as µL/mL of essential oils against JM109 Escherichia coli parental cells, and its derivate ampicillin-, ciprofloxacin-, and gentamicin-resistant cells. The values represent the mean of three independent triplicate experiments with standard error and mean. The OD_600_ values of the oil-free positive and negative controls were 1.85 ± 0.12 and 0.035 ± 0.01, respectively.

Essential Oils	JM109				Amp^r^				Cip^r^				Gen^r^			
	MIC		MBC		MIC		MBC		MIC		MBC		MIC		MBC	
	Mean	SD	Mean	SD	Mean	SD	Mean	SD	Mean	SD	Mean	SD	Mean	SD	Mean	SD
*Clinopodium nepeta*	0.966	0.057	1.000	0.000	0.633	0.058	0.800	0.000	0.867	0.115	1.000	0.058	0.900	0.000	0.900	0.058
*Citrus bergamia*	6.333	0.577	5.667	0.577	4.667	0.577	4.667	0.577	10.000	0.000	10.000	0.000	10.000	0.000	10.000	0.000
*Citrus limon*	5.000	0.000	5.000	0.000	2.000	0.000	2.000	0.000	8.333	1.528	10.000	0.000	3.000	0.000	3.000	0.000
*Citrus reticulata*	2.333	0.577	2.000	0.000	2.000	0.000	2.000	0.000	2.667	1.155	2.333	0.577	3.000	1.000	4.667	0.577
*Foeniculum vulgare*	0.400	0.000	0.400	0.000	0.367	0.058	0.400	0.000	0.567	0.058	0.600	0.000	0.333	0.058	0.400	0.000
*Laurus nobilis*	2.333	0.577	3.000	0.000	2.000	0.000	2.000	0.000	2.000	0.000	2.000	0.000	2.000	0.000	2.000	0.000
*Myrtus communis*	4.333	0.577	5.000	0.000	3.667	0.577	5.000	0.000	10.000	0.000	10.000	0.000	5.667	0.577	6.333	0.577
*Origanum vulgare*	0.300	0.000	0.300	0.000	0.300	0.000	0.300	0.000	0.300	0.000	0.300	0.000	0.300	0.000	0.300	0.000
*Salvia officinalis*	10.000	0.000	10.000	0.000	2.000	0.000	2.000	0.000	4.333	0.577	6.333	1.155	4.000	0.000	4.000	0.000
*Salvia rosmarinus*	2.000	0.000	2.333	0.577	2.000	0.000	2.000	0.000	6.333	0.577	7.000	0.000	2.333	0.577	3.000	0.000

**Table 2:** List of the major components characterized by GC–MS in the essential oils (relative abundance ≥ 1%).

Essential Oil	Component Name	%
*Clinopodium nepeta* (L.) *Kuntze*	piperitone oxide	34.28
	piperitenone oxide	18.23
	(+)-limonene	15.80
	(+)-pulegone	13.75
	menthone	8.32
	isolegylacetate	3.64
	1-terpine-4-ol	1.40
	(+)-neomenthol	1.37
	*β*-pinene	1.22
*Citrus bergamia, (Risso & Poit.)*	(+)-limonene	38.88
	lynalyl acetate	34.28
	(+)-linalool	11.54
	α-terpinene	6.79
	*β*-pinene	5.49
	*α*-pinene	1.22
*Citrus limon* (L.) *Osbeck*	(+)-limonene	74.41
	α-terpinene	11.91
	*β*-pinene	4.34
	α-terpineol	3.01
	*α*-terpinolene	1.67
	1-terpine-4-ol	1.26
*Citrus reticulata (Blanco)*	(+)-sabinene	50.91
	(+)-linalool	18.27
	*α*-phellandrene	6.54
	*β*-cis-ocimene	6.45
	(+)-limonene	5.04
	*β*-myrcene	2.37
	*β*-pinene	2.35
	*α*-pinene	1.93
	*β*-citronella	1.44
	*α*-terpinolene	1.37
	α-terpinene	1.14
*Foeniculum vulgare* subsp. *piperitum (Ucria) Bég.*	estragole	45.33
	*α*-pinene	14.71
	anethal	14.54
	fenchone	11.24
	*α*-limonene	8.49
	*α*-phellandrene	2.51
	*β*-pinene	1.65
	*β*-myrcene	1.05
*Laurus nobilis* L.	eucalyptol	56.61
	(+)-sabinene	15.74
	(+)-linalool	7.38
	terpinyl acetate	6.48
	*α*-pinene	5.65
	methyleugenol	1.51
	1-terpine-4-ol	1.29
*Myrtus communis* L.	eucalyptol	33.04
	(−)-myrtenylacetate	17.04
	*α*-pinene	12.33
	(+)-limonene	10.81
	(+)-linalool	10.43
	lynalyl acetate	3.88
	geraniol acetate	1.88
	α-terpineol	2.10
	*β*-ocimene	1.58
	*α*-phellandrene	1.41
	o-cymene	1.41
	terpinolene	1.10
	terpinene	1.09
*Origanum vulgare* L. subsp. *viridulum (Martrin-Donos) Nyman*	p-thymol	47.31
	terpinene	18.52
	p-cymene	11.78
	*β*-caryophyllene	4.88
	*β*-myrcene	3.76
	carvacrol	3.52
	terpinolene	3.18
	*α*-thujene (origanene)	2.73
	*α*-pinene	1.23
*Salvia officinalis* L.	eucalyptol	23.70
	(−)-α-thujone	24.14
	*β*-pinene	15.10
	(−)-camphor	9.59
	α-humulene	5.54
	(−)-*β*-thujone	4.35
	*α*-pinene	3.99
	(−)-*β*-caryophyllene	2.85
	*β*-myrcene	2.26
	camphene	1.88
	(+)-sabinene	1.13
*Salvia rosmarinus Spenn*	eucalyptol	49.29
	*α*-pinene	22.84
	*β*-pinene	9.26
	camphene	6.70
	(−)-camphor	3.66
	isoborneol	2.28
	*β*-myrcene	1.79
	(−)-*β*-caryophyllene	1.17

## Data Availability

Methyation data are available in the Laboratory of Biology and Genetics of Microorganisms, Department of Biology, Ecology and Earth Sciences, University of Calabria at the link: https://www.unical.it/portale/strutture/dipartimenti_240/dibest/ricerca/lab/geneticamicrobiologi/ (accessed on 6 June 2021).

## References

[1] Cragg G.M., Newman D.J. (2013). Natural Products: A Continuing Source of Novel Drug Leads. Biochim. Biophys. Acta.

[2] Sharifi-Rad J., Sureda A., Tenore G.C., Daglia M., Sharifi-Rad M., Valussi M., Tundis R., Sharifi-Rad M., Loizzo M.R., Ademiluyi A.O. (2017). Biological Activities of Essential Oils: From Plant Chemoecology to Traditional Healing Systems. Molecules.

[3] Lautié E., Russo O., Ducrot P., Boutin J.A. (2020). Unraveling Plant Natural Chemical Diversity for Drug Discovery Purposes. Front. Pharmacol..

[4] Dini I., Laneri S. (2021). Spices, Condiments, Extra Virgin Olive Oil and Aromas as not only Flavorings, but Precious Allies for our Wellbeing. Antioxidants.

[5] Gavahian M., Chu Y.H., Lorenzo J.M., Mousavi Khaneghah A., Barba F.J. (2020). Essential Oils as Natural Preservatives for Bakery Products: Understanding the Mechanisms of Action, Recent Findings, and Applications. Crit. Rev. Food Sci. Nutr..

[6] Falleh H., Ben Jemaa M., Saada M., Ksouri R. (2020). Essential Oils: A Promising Eco-Friendly Food Preservative. Food Chem..

[7] Valdivieso-Ugarte M., Gomez-Llorente C., Plaza-Díaz J., Gil Á. (2019). Antimicrobial, Antioxidant, and Immunomodulatory Properties of Essential Oils: A Systematic Review. Nutrients.

[8] Heghes S.C., Vostinaru O., Rus L.M., Mogosan C., Iuga C.A., Filip L. (2019). Antispasmodic Effect of Essential Oils and their Constituents: A Review. Molecules.

[9] Sandner G., Heckmann M., Weghuber J. (2020). Immunomodulatory Activities of Selected Essential Oils. Biomolecules.

[10] Barra A. (2009). Factors Affecting Chemical Variability of Essential Oils: A Review of Recent Developments. Nat. Prod. Commun..

[11] Wojtunik-Kulesza K.A., Kasprzak K., Oniszczuk T., Oniszczuk A. (2019). Natural Monoterpenes: Much More than Only a Scent. Chem. Biodivers..

[12] Abers M., Schroeder S., Goelz L., Sulser A., St Rose T., Puchalski K., Langland J. (2021). Antimicrobial Activity of the Volatile Substances from Essential Oils. BMC Complement Med. Ther..

[13] Bora H., Kamle M., Mahato D.K., Tiwari P., Kumar P. (2020). Citrus Essential Oils (CEOS) and their Applications in Food: An Overview. Plants.

[14] Kaya D.A., Ghica M.V., Dănilă E., Öztürk Ş., Türkmen M., Albu Kaya M.G., Dinu-Pîrvu C.E. (2020). Selection of Optimal Operating Conditions for Extraction of Myrtus Communis L. Essential Oil by the Steam Distillation Method. Molecules.

[15] Ni Z.-J., Wang X., Shen Y., Thakur K., Han J., Zhang J.-G., Hu F., Wei Z.-J. (2021). Recent Updates on the Chemistry, Bioactivities, Mode of Action, and Industrial Applications of Plant Essential Oils. Trends Food Sci. Technol..

[16] Bakkali F., Averbeck S., Averbeck D., Idaomar M. (2008). Biological Effects of Essential Oils—A Review. Food Chem. Toxicol..

[17] Dhifi W., Bellili S., Jazi S., Bahloul N., Mnif W. (2016). Essential Oils’ Chemical Characterization and Investigation of Some Biological Activities: A Critical Review. Medicines.

[18] Fung F., Wang H.S., Menon S. (2018). Food Safety in the 21st Century. Biomed. J..

[19] Mith H., Duré R., Delcenserie V., Zhiri A., Daube G., Clinquart A. (2014). Antimicrobial Activities of Commercial Essential Oils and their Components against Food-Borne Pathogens and Food Spoilage Bacteria. Food Sci. Nutr..

[20] Bouarab Chibane L., Degraeve P., Ferhout H., Bouajila J., Oulahal N. (2019). Plant Antimicrobial Polyphenols as Potential Natural Food Preservatives. J. Sci. Food Agric..

[21] Larsson D.G.J., Flach C.F. (2021). Antibiotic Resistance in the Environment. Nat. Rev. Microbiol..

[22] Gutiérrez-Del-Río I., López-Ibáñez S., Magadán-Corpas P., Fernández-Calleja L., Pérez-Valero Á., Tuñón-Granda M., Miguélez E.M., Villar C.J., Lombó F. (2021). Terpenoids and Polyphenols as Natural Antioxidant Agents in Food Preservation. Antioxidants.

[23] Álvarez-Martínez F.J., Barrajón-Catalán E., Encinar J.A., Rodríguez-Díaz J.C., Micol V. (2020). Antimicrobial Capacity of Plant Polyphenols against Gram-positive Bacteria: A Comprehensive Review. Curr. Med. Chem..

[24] Álvarez-Martínez F.J., Barrajón-Catalán E., Herranz-López M., Micol V. (2021). Antibacterial Plant Compounds, Extracts and Essential Oils: An Updated Review on their Effects and Putative Mechanisms of Action. Phytomedicine.

[25] Pattnaik S., Subramanyam V.R., Bapaji M., Kole C.R. (1997). Antibacterial and Antifungal Activity of Aromatic Constituents of Essential Oils. Microbios.

[26] Kalemba D., Kunicka A. (2003). Antibacterial and Antifungal Properties of Essential Oils. Curr. Med. Chem..

[27] Fournomiti M., Kimbaris A., Mantzourani I., Plessas S., Theodoridou I., Papaemmanouil V., Kapsiotis I., Panopoulou M., Stavropoulou E., Bezirtzoglou E.E. (2015). Antimicrobial Activity of Essential Oils of Cultivated Oregano (*Origanum vulgare*), Sage (*Salvia officinalis*), and Thyme (*Thymus vulgaris*) against Clinical Isolates of *Escherichia Coli*, Klebsiella Oxytoca, and Klebsiella Pneumoniae. Microb. Ecol. Health Dis..

[28] Tariq S., Wani S., Rasool W., Shafi K., Bhat M.A., Prabhakar A., Shalla A.H., Rather M.A. (2019). A Comprehensive Review of the Antibacterial, Antifungal and Antiviral Potential of Essential Oils and their Chemical Constituents against Drug-Resistant Microbial Pathogens. Microb. Pathog..

[29] Lambert R.J., Skandamis P.N., Coote P.J., Nychas G.J. (2001). A Study of the Minimum Inhibitory Concentration and Mode of Action of Oregano Essential Oil, Thymol and Carvacrol. J. Appl. Microbiol..

[30] Chandra H., Bishnoi P., Yadav A., Patni B., Mishra A.P., Nautiyal A.R. (2017). Antimicrobial Resistance and the Alternative Resources with Special Emphasis on Plant-Based Antimicrobials—A Review. Plants.

[31] Khameneh B., Iranshahy M., Soheili V., Fazly Bazzaz B.S. (2019). Review on Plant Antimicrobials: A Mechanistic Viewpoint. Antimicrob. Resist. Infect. Control..

[32] Alibi S., Ben Selma W., Ramos-Vivas J., Smach M.A., Touati R., Boukadida J., Navas J., Ben Mansour H. (2020). Anti-Oxidant, Antibacterial, Anti-Biofilm, and Anti-Quorum Sensing Activities of Four Essential Oils against Multidrug-Resistant Bacterial Clinical Isolates. Curr. Res. Transl. Med..

[33] Sharifi A., Mohammadzadeh A., Zahraei Salehi T., Mahmoodi P. (2018). Antibacterial, Antibiofilm and Antiquorum Sensing Effects of Thymus Daenensis and Satureja Hortensis Essential Oils against Staphylococcus Aureus Isolates. J. Appl. Microbiol..

[34] Zhang D., Gan R.Y., Zhang J.R., Farha A.K., Li H.B., Zhu F., Wang X.H., Corke H. (2020). Antivirulence Properties and Related Mechanisms of Spice Essential Oils: A Comprehensive Review. Compr. Rev. Food Sci. Food Saf..

[35] Reichling J. (2020). Anti-Biofilm and Virulence Factor-Reducing Activities of Essential Oils and Oil Components as a Possible Option for Bacterial Infection Control. Planta Med..

[36] Sandoval-Motta S., Aldana M. (2016). Adaptive Resistance to Antibiotics in Bacteria: A Systems Biology Perspective. Wiley Interdiscip. Rev. Syst. Biol. Med..

[37] Hao Y., Kang J., Yang R., Li H., Cui H., Bai H., Tsitsilin A., Li J., Shi L. (2021). Multidimensional Exploration of Essential Oils Generated Via Eight Oregano Cultivars: Compositions, Chemodiversities, and Antibacterial Capacities. Food Chem..

[38] Pereira C., Barros L., Ferreira I.C. (2015). A Comparison of the Nutritional Contribution of Thirty-Nine Aromatic Plants Used as Condiments and/or Herbal Infusions. Plant Foods Hum. Nutr..

[39] Carlson J.L., Erickson J.M., Lloyd B.B., Slavin J.L. (2018). Health Effects and Sources of Prebiotic Dietary Fiber. Curr. Dev. Nutr..

[40] Ahmed W., Rashid S. (2019). Functional and Therapeutic Potential of Inulin: A Comprehensive Review. Crit. Rev. Food Sci. Nutr..

[41] Thielmann J., Muranyi P., Kazman P. (2019). Screening Essential Oils for their Antimicrobial Activities against the Foodborne Pathogenic Bacteria Escherichia Coli and Staphylococcus Aureus. Heliyon.

[42] Puškárová A., Bučková M., Kraková L., Pangallo D., Kozics K. (2017). The Antibacterial and Antifungal Activity of Six Essential Oils and their Cyto/Genotoxicity to Human HEL 12469 Cells. Sci. Rep..

[43] Arantes S.M., Piçarra A., Guerreiro M., Salvador C., Candeias F., Caldeira A.T., Martins M.R. (2019). Toxicological and Pharmacological Properties of Essential Oils of *Calamintha Nepeta*, *Origanum Virens* and *Thymus Mastichina* of Alentejo (Portugal). Food Chem. Toxicol..

[44] Gormez A., Bozari S., Yanmis D., Gulluce M., Sahin F., Agar G. (2015). Chemical Composition and Antibacterial Activity of Essential Oils of Two Species of Lamiaceae against Phytopathogenic Bacteria. Pol. J. Microbiol..

[45] Dadalioglu I., Evrendilek G.A. (2004). Chemical Compositions and Antibacterial Effects of Essential Oils of Turkish Oregano (*Origanum minutiflorum*), Bay Laurel (*Laurus nobilis*), Spanish Lavender (*Lavandula Stoechas* L.), and Fennel (*Foeniculum vulgare*) on Common Foodborne Pathogens. J. Agric. Food Chem..

[46] Ruberto G., Baratta M.T., Deans S.G., Dorman H.J. (2000). Antioxidant and Antimicrobial Activity of *Foeniculum vulgare* and Crithmum Maritimum Essential Oils. Planta Med..

[47] Pellegrini M., Ricci A., Serio A., Chaves-López C., Mazzarrino G., D’Amato S., Lo Sterzo C., Paparella A. (2018). Characterization of Essential Oils Obtained from Abruzzo Autochthonous Plants: Antioxidant and Antimicrobial Activities Assessment for Food Application. Foods.

[48] Cetin B., Ozer H., Cakir A., Polat T., Dursun A., Mete E., Oztürk E., Ekinci M. (2010). Antimicrobial Activities of Essential Oil and Hexane Extract of Florence Fennel [*Foeniculum vulgare* Var. Azoricum (Mill.) Thell.] against Foodborne Microorganisms. J. Med. Food.

[49] Napoli E., Giovino A., Carrubba A., How Yuen Siong V., Rinoldo C., Nina O., Ruberto G. (2020). Variations of Essential Oil Constituents in Oregano (*Origanum vulgare* subsp. *viridulum* (= *O. heracleoticum*) over Cultivation Cycles. Plants.

[50] Novak J., Lukas B., Franz C. (2010). Temperature Influences Thymol and Carvacrol Differentially in *Origanum* spp.(Lamiaceae). J. Essent. Oil Res..

[51] Giuliani C., Maggi F., Papa F., Maleci Bini L. (2013). Congruence of Phytochemical and Morphological Profiles along an Altitudinal Gradient in Origanum vulgare ssp. vulgare from Venetian Region (NE Italy). Chem. Biodivers..

[52] Kokkini S., Karousou R., Dardioti A., Krigas N., Lanaras T. (1997). Autumn essential oils of Greek Oregano. Phytochemistry.

[53] Carrubba A., Catalano C., Lichtfouse E. (2009). Essential Oil Crops for Sustainable Agriculture—A review. Climate Change, Intercropping, Pest Control and Beneficial Microorganisms.

[54] Gullberg E., Cao S., Berg O.G., Ilbäck C., Sandegren L., Hughes D., Andersson D.I. (2011). Selection of Resistant Bacteria at Very Low Antibiotic Concentrations. PLoS Pathog..

[55] Unusan N. (2020). Essential Oils and Microbiota: Implications for Diet and Weight Control. Trend Food Sci. Technol..

[56] Sánchez-Romero M.A., Casadesús J. (2020). The Bacterial Epigenome. Nat. Rev. Microbiol..

